# Ultrasound and Unsupervised Segmentation-Based Gesture Recognition for Smart Device Unlocking

**DOI:** 10.3390/s25206408

**Published:** 2025-10-17

**Authors:** Xiaojuan Wang, Mengqiao Li

**Affiliations:** 1 College of Mathematics and Computer Science, Northwest Minzu University, Lanzhou 730030, China; 2 Gansu Provincial Engineering Research Center of Multi-Modal Artificial Intelligence, Northwest Minzu University, Lanzhou 730030, China; 3 College of Computer Science and Engineering Northwest Normal University, Lanzhou 730070, China; 13919027871@163.com

**Keywords:** acoustic sensing, unsupervised segmentation, sign language recognition, smart device unlocking

## Abstract

A smart device unlocking scheme based on ultrasonic gesture recognition is proposed, allowing users to unlock their devices by customizing the unlock code through gesture movements. This method utilizes ultrasound to detect multiple consecutive gestures, identifying micro-features within these gestures for authentication. To enhance recognition accuracy, an unsupervised segmentation algorithm is employed to accurately segment the gesture feature region and extract the time-frequency domain data of the gestures. Additionally, two-stage data enhancement techniques are applied to generate user-specific data based on a small sample size. Finally, the user-specific model is deployed to mobile devices via transfer learning for on-device, real-time inference. Experimental validation on a commercial smartphone (Redmi K50) demonstrates that the entire authentication pipeline, from signal acquisition to decision, processes 8 types of gestures in a sequence in sequence in approximately 1.2 s, with the core model inference taking less than 50 milliseconds. This ensures that the raw biometric data (ultrasonic echoes) and the recognition results never leave the user’s device during authentication, thereby safeguarding privacy. It is important to note that while model training is performed offline on a server to leverage greater computational resources for personalization, the deployed system operates fully in real time on the edge device. Experimental results demonstrate that our system achieves accurate and robust identity verification, with an average five-fold cross-validation accuracy rate of up to 93.56%, and it shows robustness across different environments.

## 1. Introduction

As Internet of Things (IoT) technology rapidly advances, an increasing number of intelligent devices and sensors are becoming connected within this vast network. Consequently, traditional human–computer interaction methods, which rely on simple mechanical devices like keyboards and mice, are gradually becoming obsolete. These methods are being replaced by a range of emerging human–computer interaction technologies [1]. These emerging technologies not only enhance the efficiency of interactions between users and intelligent terminals but also greatly enrich the interaction mode, making human–computer interaction more intelligent, efficient, and diversified [2]. In recent years, there has been a significant increase in the development and implementation of human–computer intelligent feedback systems and natural language input schemes. This emergence signals that human–computer interaction technology is advancing into a new stage of development [3]. Among the various emerging methods of interaction, gesture-based interaction has garnered considerable attention due to its widespread use and applicability. By detecting user gestures, intelligent devices can accurately interpret actions and provide appropriate responses, facilitating a more natural and intuitive interaction experience [4,5,6]. Research in gesture interaction has yielded impressive results, enhancing the efficiency of communication between humans and objects, as well as among objects themselves. This progress has profound implications for fields such as artificial intelligence and communication systems. For example, gesture interaction technology is now widely applied in smart home environments [7], smart medical settings [8], and smart driving scenarios [9,10], offering users a more convenient and efficient operation experience. As a result, exploring more intelligent and efficient wireless gesture-based human–computer interaction systems has become a priority in this field. Such advancements will not only propel the development of human–computer interaction technology but also provide strong technical support for innovations in the Internet of Things and artificial intelligence.

There are two main types of gesture recognition techniques: single gesture recognition and continuous gesture recognition [11,12]. Compared to single gesture recognition, continuous gesture recognition offers several significant advantages [12]. Firstly, continuous gesture recognition can capture and understand a user’s true intentions more accurately. In everyday life, people often convey complex thoughts or instructions through a series of consecutive gestures rather than isolated ones [13]. This technology can interpret a sequence of gestures as a whole, allowing for a more precise understanding of the user’s complete intent and improving recognition accuracy. Secondly, continuous gesture recognition provides a more natural and fluid user interaction experience. Users do not need to deliberately stop and separate their gestures; they can move continuously according to their own habits and rhythms. This approach makes human–computer interaction more aligned with everyday behavior, enhancing intuitiveness and ease of use [14]. Additionally, continuous gesture recognition is more flexible and scalable. As technology continues to advance, the accuracy and efficiency of this method will improve, supporting a wider variety of gestures and more complex interaction scenarios. Furthermore, it can be integrated with other interaction modes (such as speech and eye movement) to create a multimodal interaction system, which further enhances the user experience and interaction efficiency [15].

Gesture recognition strategies at this stage are divided into two main categories [16]. One is wearable device-based gesture recognition systems. These systems rely on specific devices worn by the user, such as myoelectric patches, gloves, or other sensor devices. They recognize and analyze gestures by capturing the user’s hand movements and converting them into digital signals. The other is gesture sensing systems for contactless devices. Unlike wearable devices, these systems do not require the user to wear any additional equipment. Instead, they utilize advanced camera, sensor, or radar technologies to capture and analyze the user’s gestures without physical contact. This allows for gesture recognition and response without the need for devices worn by the user.

Jiang et al. developed a stretchable electronic skin (E-skin) [17] that integrates pressure and contact sensors for accurate recognition of complex gestures. They classified the gestures using machine learning techniques—specifically, Support Vector Machines (SVM)—achieving an impressive accuracy of 98%. This technology can be utilized for gesture recognition and intelligent control. Duan et al. [18] applied the discrete wavelet transform (DWT) technique to extract features from filtered surface electromyographic (sEMG) signals, proposing a high-precision gesture recognition system. This system achieved a recognition accuracy of over 93% in complex environments, enhancing the control of human–computer interaction devices based on gestures. Yang et al. [19] introduced a multistream residual network for recognizing dynamic hand movements based on sEMG signals by analyzing the electrical activity of muscles to identify hand gestures. Their approach combines a residual model with a convolutional short-term memory model into a unified framework. This architecture extracts spatio-temporal features both globally and deeply while integrating feature fusion to retain essential information. While wearable-based gesture recognition systems can achieve high accuracy, they may not be the best solution for continuous gesture recognition due to the high cost of wearable devices and the discomfort associated with wearing them.

As the number of mobile devices connected to the Internet and the Internet of Things (IoT) continues to grow, user privacy and security have become increasingly significant topics in human–computer interaction (HCI) research. Mobile devices are typically secured with screen locks, and user identity is verified through methods such as passwords and fingerprints [20]. While there are various methods for recognizing user characteristics, finding an authentication approach that combines low power consumption, high availability, and strong security remains a challenging task in pattern recognition. Recent groundbreaking research has explored the use of ultrasonic signals transmitted and received by smartphones to capture behavioral patterns for non-invasive activity recognition. This includes the tracking of fine hand movements [21], lip recognition [22], and even blinking recognition [23]. Imagine a system that allows users to unlock a device by gesturing for numbers 0–9 or performing a custom gesture in front of the mobile device. To unlock the device, users must provide the correct sequence of gestures. Importantly, even if different users perform the same gesture, they cannot unlock the device unless they provide the correct sequence specific to their own gestures. This gesture unlocking system creates a large training dataset based on a small number of samples submitted by the user. It can quickly and accurately recognize each gesture from a defined set and calibrate the involved timing. The system is not dependent on specific hardware [24,25] and has low environmental requirements for operation. The key concept of gesture authentication lies in recognizing the differences among gestures performed by different users. Variations in the speed and amplitude of gestures can occur due to differences in skeletal muscle structure and personal lifestyle habits. While individual gestures may differ only slightly, the differences between multiple consecutive gestures are more significant. This is because each user tends to maintain relatively consistent durations and amplitude variations for different gestures, along with independent onset and release actions. As a result, it becomes feasible to authenticate users and unlock the device using a sequence of multiple consecutive gestures. The adoption of this offline training strategy can effectively handle the computational requirements of user-specific data augmentation and model optimization, which do not have high time requirements. After training, the model is deployed on the user’s mobile device, with the inference process on the device (including signal acquisition, processing, segmentation, feature extraction, and gesture classification) comprehensively optimized for real-time operation. It can be verified within 1.2 s, indicating that the system has good real-time capabilities, ensuring that the real-time requirements of user experience applications are met. The main advantages of SonarGS compared to existing ultrasonic or gesture authentication systems include the following:

1. Accurate extraction of continuous gesture features using unsupervised segmentation methods (Felzenszwalb + DBSCAN) rather than depending on fixed time windows or thresholds;
2. A two-stage data enhancement strategy for user-personalized micro-features, which enhances the model’s ability to generalize, even with small sample sizes;
3. A mobile deployment scheme based on transfer learning, which ensures that security features, such as passwords (model weights), are not transmitted over the network.

This paper propose SonarGS, a smart device unlocking system that utilizes gesture recognition based on ultrasound and unsupervised segmentation. First, we transmit ultrasonic signalsat a sampling rate of 20 kHz using a transceiver developed by WeChat. The ultrasonic echoes of the gestures are then smoothed through basic filtering, and background noise present in dynamic environments is eliminated using D-Doppler technology to address frame activity anomalies caused by hardware. We apply an unsupervised segmentation algorithm, along with a smoothing algorithm, to segment and refine the gesture features obtained from each Doppler frequency-shift image. Additionally, a Doppler feature extraction algorithm accurately captures the gesture’s time–frequency-domain information and identifies gesture feature regions. A few shot-based gesture training dataset is created using a two-stage data enhancement method. Finally, the processed gesture feature maps are input into the authentication module, where a dedicated classification model is trained for each user. We also employ transfer learning via a mobile device using the Paddle Lite deep learning framework to establish thresholds and verify whether the recognition results are accurate, thereby determining the user’s identity.

## 2. Ultrasound-Based Gesture Perception Theory and Methodology

### 2.1. Transmission and Reception of Signals

Ultrasonic gesture recognition technology is an advanced interaction method that utilizes ultrasonic signals with frequencies higher than 20 kHz to detect and interpret gesture movements. The range of human hearing typically falls between 20 Hz and 20 kHz [26], so ultrasonic gesture recognition systems operate above this threshold—usually at frequencies exceeding 20 kHz. This ensures that the signals do not interfere with the user’s auditory experience while achieving high-precision gesture detection. These ultrasonic gesture sensing techniques generally use a speaker as a transmitter to emit ultrasonic signals and a high-precision microphone as a receiver to capture ultrasonic echoes [27]. The echoes are sampled by microphones positioned strategically within the environment [28]. By employing complex signal processing and analysis techniques—including time difference measurement, phase-change resolution, and frequency offset detection—the system can accurately identify various features of gestures, such as their position, shape, velocity, and direction [29].

**Figure 1:**
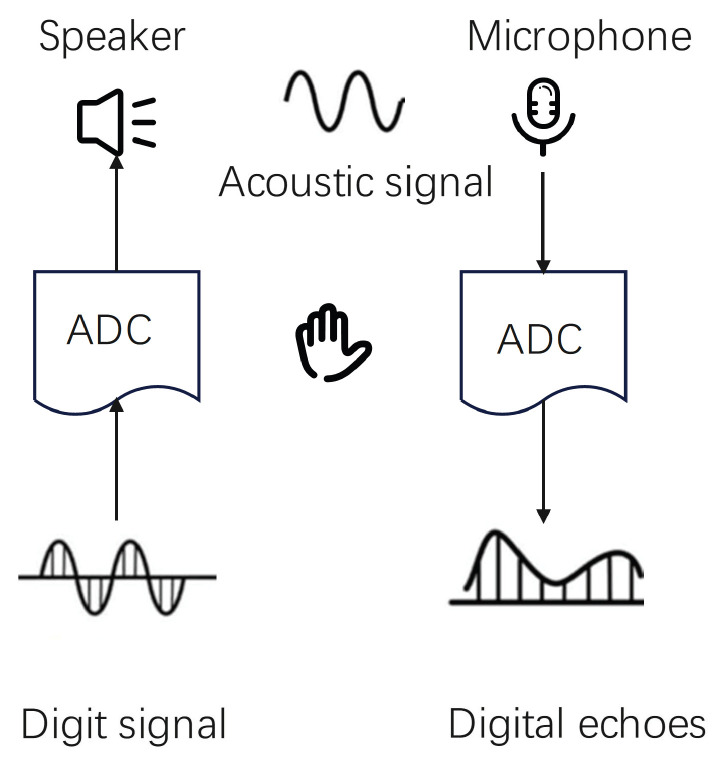
Transmission and reception of signals.

Ultrasonic signal transceivers can be categorized into two main types based on their structural and functional characteristics: single-speaker microphones and microphone arrays [16]. A single speaker consists of a single audio output unit. However, due to having only one sound-generating unit, it may have limitations in terms of stereo sound, directivity, and coverage. As illustrated in Figure 1, mono systems utilize a single speaker to emit ultrasonic signals, which are typically of a single frequency or modulated signals. In contrast, a microphone array [30] is an audio input device composed of multiple microphones arranged in a specific pattern. This setup is primarily used for sound acquisition and localization, which can significantly enhance the quality and accuracy of sound signal capture. By operating multiple microphones simultaneously, a microphone array can achieve improvements in enhancement, noise reduction, and beam formation, allowing for clear and precise sound signals, even in complex environments.

### 2.2. Signal Generation

Ultrasonic sensing technology is widely used in various applications, including gesture recognition, distance measurement, and environmental sensing. The three most commonly used types of ultrasonic signals are monophonic ultrasound signals [31], chirp signals [32], and Frequency-Hopping Spread Spectrum (FHSS) signals [33].

Monophonic ultrasonic signals are primarily used for basic distance measurement and obstacle detection due to their simplicity and straightforward nature. A monophonic ultrasound refers to an ultrasonic signal that has a fixed and constant frequency. Such signals are commonly used for applications such as distance measurement, speed measurement, and simple obstacle detection.

Chirp signals [34] are ultrasonic signals with frequencies that increase or decrease linearly over time. They provide excellent time and frequency resolution, making them ideal for accurately recognizing gesture direction and speed. This characteristic allows chirp signals to provide richer information, since changes in frequency can reflect the relative motion between an object (such as a gesture) and the sensor.

An FHSS signal improves resistance to interference and enhances the stability of signal transmission by rapidly switching between different frequencies. This allows for high recognition accuracy, even in complex environments. In a dual-channel system, two speakers can transmit signals either separately or simultaneously, utilizing principles of phase difference or time difference. This not only enables two-dimensional (2D) or even three-dimensional (3D) gesture recognition but also effectively isolates multipath reflections using multi-channel signal processing technology. As a result, the system’s recognition accuracy and stability are significantly enhanced.

**Figure 2:**
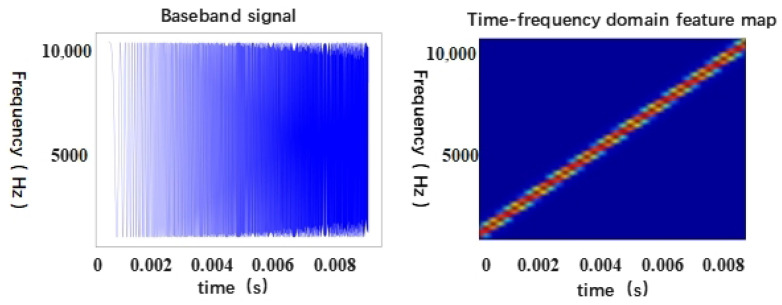
Baseband signals and spectrograms of FMCW.

A common application of acoustic gesture sensing is Frequency-Modulated Continuous Wave (FMCW) ultrasonic wireless ranging [35]. FMCW radar is a type of continuous wave radar in which the frequency of the transmitted signal varies continuously over time. This radar technology calculates the distance and speed of a target by comparing the frequency difference between the transmitted signal and the received echo signal. As illustrated in Figure 2, an FMCW radar transmits a series of continuous FM waves and receives reflected signals from a target. The frequency of the transmitted wave varies over time according to a specific modulation pattern. The frequency difference between the returned signal and the transmitted signal contains valuable information about the distance and speed of the target.

### 2.3. Pure-Tone Ultrasound and the Doppler Effect

The Doppler effect is the change in the frequency of a wave that occurs when the emitter, receiver (or both) is are in relative motion. In acoustics, this effect refers to the alteration in the frequency of sound due to the relative movement of the sound source or the listener. When the source and listener are close together, the frequency of the sound increases, while the frequency decreases when they are farther apart. If the frequency of the sound source remains constant, an observer moving away from the source will receive a lower frequency and a longer wavelength. Conversely, when the observer approaches the source, they will perceive a higher frequency and a shorter wavelength. As indicated in (1), the frequency received by the observer is *f*, the propagation speed of the wave in the medium is *c*, the speed of the observer is $v_{h}$, the speed of the wave source is $v_{w}$, and the original frequency emitted by the wave source is $f_{s}$.

(1) $$f=\left(\frac{c\pm v_{h}}{c\mp v_{w}}\right)\cdot f_{s}.$$

**Figure 3:**
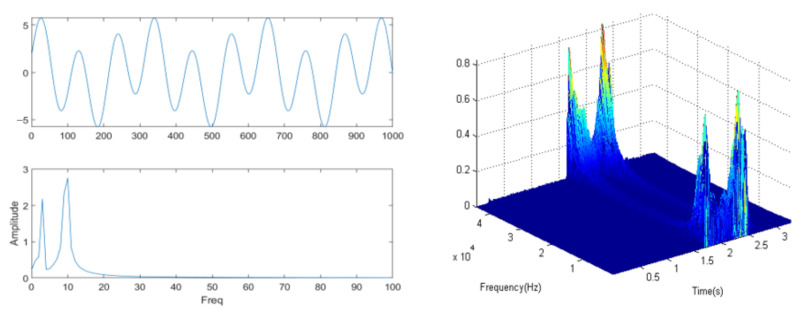
Schematic diagram of short-time Fourier transform.

The Doppler effect causes changes in the frequency of ultrasonic detector waves due to gesture movements. The frequency shift resulting from these gestures is analyzed over time using Short-Time Fourier Transform (STFT). STFT is a widely used tool in signal processing and spectral analysis that allows for the localization of frequency-domain characteristics within signals. Unlike the traditional Fourier transform, STFT enables us to observe how a signal evolves over time. This process is illustrated in Figure 3.

STFT involves the sampling of a signal into equal-length observation frames using a matrix windowing function, followed by the performance of a Fourier transform on each sampled frame. By sliding the window along the time axis, we can capture the local characteristics of the signal in both time and frequency domains. First, we gather the signal data related to the gesture action. It is essential to preprocess this signal, which includes noise removal and filtering, to ensure that the resulting spectrum can be analyzed more accurately. The signal is then divided into smaller segments based on the window function; these segments are referred to as sampling frames. This segmentation is important, as it helps preserve the time-domain information of the signal while allowing for spectral analysis of each frame, thereby minimizing the effects of spectral leakage. Next, a Fourier transform is applied to the windowed data of each frame to represent that frame in the frequency domain. Finally, all the sampled frames are organized according to their time sequence, resulting in the short-time Fourier matrix. The mathematical representation of the short-time Fourier transform is shown in Equation (2).

(2) $$X\left(t,f\right)=\int x\left(\tau \right)w\left(\tau -t\right)e^{-j2\pi f\tau }d\tau .$$

where w(τ−t) is a window function used to split the signal into small chunks in time. Commonly used window functions include a rectangular window, Hanning window, and so on. X(t,f) represents the signal components at time *t* and frequency *f*. Since computers can only handle discrete data, the discrete form of STFT can be obtained by converting the integral into a discrete sum, as shown in (3):

(3) $$X\left(m,n\right)=\sum_{k=0}^{N-1}x\left(n+k\right)\cdot w\left(k\right)\cdot e^{-j\frac{2\pi }{N}mk}.$$

where X(m,n) denotes the discrete STFT coefficients at the time index (*m*) and frequency index (*n*), which is the value of the input signal at the discrete time point. w(k) is the value of the window function at the discrete time point, and *N* is the length of the window.

### 2.4. FMCW-Based Gesture Ranging

Frequency Modulated Continuous Wave (FMCW) is a widely adopted technique in high-precision radar ranging systems. An FMCW signal is characterized by a frequency that increases linearly over time. The fundamental principle of FMCW-based ranging is to transmit a signal with linearly modulated frequency and analyze the frequency difference between the transmitted and received signals to estimate the target distance.

Assuming that the acoustic source is stationary and the recording device remains fixed relative to the source, the transmitted signal can be expressed as follows:

(4) $$S\left(t\right)=\cos\left(2\pi \left(f_{min}+\frac{B}{2T}\right)t\right),$$

where *S* denotes the transmit signal, $f_{min}$ is the minimum FMCW frequency, and *B* is the frequency slope.

The target of the reception is the reflected recurrent signal, the demand time of the signal is distributed, the frequency of the reception signal is given, and a difference in the frequency of the signal exists.

(5) $$L\left(t\right)=A\cos\left(2\pi \left(f_{min}+\frac{B}{2T}\left(t-t_{d}\right)\right)\left(t-t_{d}\right)\right)$$

where *A* denotes the attenuation factor and $t_{d}$ represents the time delay required for the signal to propagate from the transmitter to the receiver. The transmitted signal and the received signal are multiplied to obtain the intermediate signal S(t)*L(t). According to the trigonometric product-to-sum identity, the high-frequency components can be filtered out, yielding a mixed signal (V(t)). A Fourier transform is then applied to the mixed signal to extract the frequency difference ($f_{1F}$), which is directly related to the propagation delay and, thus, to the distance.

(6) $$f_{1F}=\frac{B}{T}t_{d}$$

(7) $$t_{d}=\frac{2d}{c}$$

The formulation resulting from the combination of (6) and (7) leads to Equation (8), which enables the estimation of the time delay based on the observed beat frequency.

(8) $$f_{1F}=\frac{2Bd}{cT}$$

Given that *c* is constant under standard conditions, the real-time distance of the gesture can be calculated accordingly by $f_{IF}$.

### 2.5. Channel Impulse Response

The Channel Impulse Response (CIR) characterizes the output of a channel in response to an input signal. In ultrasonic sensing, when an ultrasonic pulse is transmitted, the channel responds to the signal, and this response is known as channel impulse response. This response reflects the fundamental characteristics of ultrasonic signal propagation, including delay, attenuation, and multipath effects. Multipath effects refer to the interference caused by ultrasonic signals propagating through multiple distinct paths before reaching the receiver. According to wireless communication principles, the baseband signal shares a similar transmission path with the passband signal. As a result, the relationship can be expressed as R[n]=S[n]*h[n], where * denotes the convolution operator; S[n] and R[n] are the transmitted and received baseband signals, respectively; and h[n] is the channel impulse response. Currently, two main methods are employed to estimate the CIR: the Least Squares (LS) method and the Maximum Likelihood Estimation (MLE) method.

In LS estimation, let *L* denote the length of the probing frame, corresponding to the CIR length in its discrete representation. Let *P* represent the feature dimension of the training matrix, and let the total data segment length (*d*) be the sum of *P* and *L*. Let *D* denote the training data segment composed of samples $s_{1}$ to $s_{d}$. The training matrix (*M*) is constructed as a circulant matrix based on the segment (*D*). To reduce computational complexity, the CIR can be estimated via a matrix-vector multiplication involving the training matrix (*M*) and the received signal (R[n]), as shown in Equation (9):

(9) $$\left[\begin{array}{cccc} s_{1} & s_{2} & \ldots & s_{L}\\ s_{2} & s_{3} & \ldots & s_{L+1}\\ \vdots & \vdots & \ddots & \vdots \\ s_{P} & s_{P+1} & \ldots & s_{P+L-1} \end{array}\right]\left[\begin{array}{c} h_{1}\\ h_{2}\\ \vdots \\ h_{L} \end{array}\right]=\left[\begin{array}{c} r_{L+1}\\ r_{L+2}\\ \vdots \\ r_{L+P} \end{array}\right]$$

MLE is a widely used method in statistics and machine learning for parameter estimation. The core idea of MLE is to determine the parameters that maximize the likelihood (or log-likelihood) function, thereby making the observed data most probable. In MLE, the likelihood function is calculated as the product of the probability density function (or probability mass function) for a parameter over the observed data, assuming that the data is independently and identically distributed. Assuming that the received signal (R[n]) follows a Gaussian distribution, the likelihood function can be modeled as a probability density function, as shown in Equation (10):

(10) $$p\left(R\left[n\right]|h\left[n\right]\right)=\frac{1}{\sqrt{2\pi \sigma^{2}}}\exp\left(-\frac{{\left(R\left[n\right]-S\left[n\right]*h\left[n\right]\right)}^{2}}{2\sigma^{2}}\right)$$

As described in Equation (11), the overall likelihood function (L(h[n])) is the joint probability density function over all *N* observed data points:

(11) $$L\left(h\right)=\prod_{n=0}^{N}p\left(R\left[n\right]|h\left[n\right]\right)$$

## 3. Overview of the SonarGS Method

**Figure 4:**
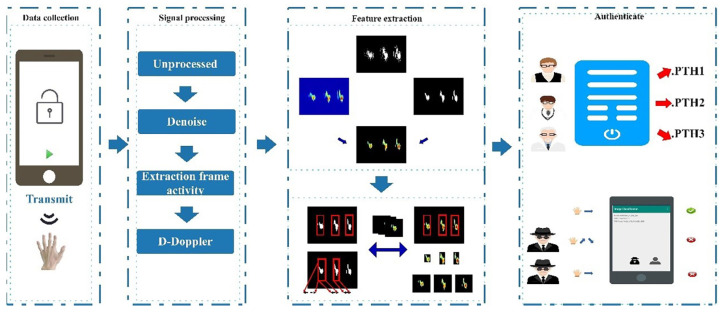
Overall architecture of the SonarGS gesture-based unlocking system.

As illustrated in Figure 4, the SonarGS intelligent device unlocking system consists of five main components: signal acquisition, data processing, data augmentation, feature extraction, and identity authentication. A gesture acquisition module was developed based on a WeChat Mini Program, which enables mobile devices to transmit ultrasonic signals at 20 kHz via the built-in speaker and simultaneously record the echo signals using the microphone. This transceiver module can be deployed on any smart mobile device capable of running the WeChat environment. During the data processing stage, SonarGS applies a combination of band-pass and notch filters to perform basic denoising of the ultrasonic echoes. Frame activation sequences are extracted using Gaussian smoothing and normalization. Environmental noise and irregular dynamic interference are mitigated through dedicated noise reduction algorithms. For data augmentation, a two-stage approach is adopted. First, user-specific gesture profiles are generated based on acquired category and time–frequency-domain information. Then, stretch–shift–stack graphical transformations are employed to augment the data. In the feature extraction stage, the SonarGS system utilizes the Felzenszwalb-DBSCAN algorithm to precisely segment the gesture feature regions from Doppler spectrograms. Subsequently, time–frequency and gesture-specific features are extracted using Doppler-based analysis methods. In the identity authentication stage, a successful verification requires the satisfaction of both the confidence threshold and a predefined sequence of gesture actions. Model training is performed on the server side, while inference and prediction tasks are executed independently on the mobile device.

### 3.1. Signal Acquisition and Processing

**Figure 5:**
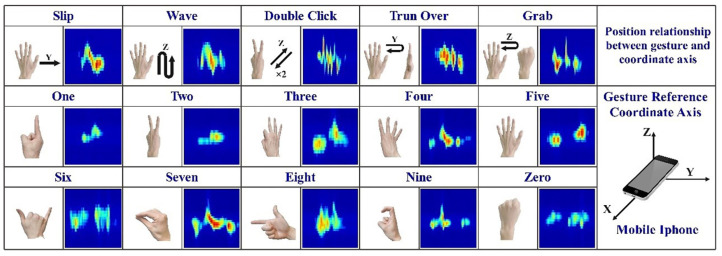
Signal acquisition interface of the SonarGS ultrasonic transceiver, illustrating 15 distinct gesture types and their corresponding Doppler effects.

SonarGS modulates a 20 kHz ultrasonic pure-tone signal and stores it within a transmitter–receiver module developed using a WeChat Mini Program. The program controls the mobile device’s speaker to emit the modulated ultrasonic waveform, while the built-in microphone records the returning echoes to capture reflected signals. The transmitter simultaneously samples the echoes during emission, enabling real-time gesture sensing. The entire acquisition process lasts for 8 s. As shown in Figure 5, the interface of the ultrasonic transmitter–receiver displays 15 gesture types and their corresponding Doppler signatures, including digit gestures from 0 to 9 and five fundamental gestures.

To suppress noise interference, SonarGS first applies a Butterworth band-pass filter to remove out-of-band noise outside the 19,000–21,000 Hz range. A notch filter is then employed to eliminate the ultrasonic carrier components within the range of 19,985–20,015 Hz. To further smooth the signal and eliminate outliers, a Gaussian filter is applied to the signal matrix. This filter attenuates values that deviate significantly from the statistical expectation, yielding a more refined spectrogram. The Gaussian filter assigns the highest weights to the central amplitudes, with weights gradually decreasing toward the periphery, forming a near-Gaussian distribution. This makes it particularly effective for the smoothing of Doppler shift images. After the initial denoising process, the system performs a Short-Time Fourier Transform (STFT) on the ultrasonic echoes to obtain a time–frequency magnitude matrix. Each sampling window is treated as a frame, and the variance of the magnitude within each frame is calculated. The variance reflects the degree of deviation from the expected value and, thus, indicates the intensity of frame variation. Given that the recording environment may contain various interferences, a differential multi-Doppler algorithm is employed to extract the gesture-specific components from the ultrasonic echoes while minimizing the influence of dynamic background noise.

### 3.2. Unsupervised Gesture Segmentation Algorithm

Preliminary experiments and analyses demonstrated that when different individuals perform continuous gestures, distinctive variations arise in initiation, release, speed, and amplitude and that these distinctions become increasingly pronounced with larger gesture sets. By capturing these fine-grained characteristics, SonarGS enables reliable user identification.

Image segmentation algorithms are effective in extracting such micro-level features. However, supervised segmentation and semantic segmentation approaches require pixel-level annotation, which is impractical in real-world scenarios and unsuitable for real-time human–computer interaction. Given the significant contrast between feature and non-feature regions in Doppler spectrograms, this study employs an unsupervised segmentation method to isolate gesture regions, followed by Doppler-based algorithms to extract temporal-frequency features of consecutive gestures across users.

The Felzenszwalb superpixel segmentation algorithm is adopted to address this problem, with three tunable parameters—scale, sigma, and minimum size—optimizing segmentation performance. The scale and minimum size control the segmentation threshold, determining which regions can be treated as independent segments, while sigma regulates sensitivity to color variations. A larger sigma value reduces sensitivity to minor variations, thereby preventing over-segmentation. The two-stage algorithm dynamically adjusts these parameters based on pseudo-labels derived from gesture amplitudes: larger amplitudes indicate salient features and require larger parameter values, while moderate or smaller amplitudes apply appropriately scaled parameters for refined segmentation. Each pixel in the image is treated as a node in a graph, represented as $C_{k}={\left[R_{k},G_{k},B_{k},x_{k},y_{k}\right]}^{T}$, where $C_{k}$ contains the color and spatial information of the pixel.The Felzenszwalb algorithm constructs image superpixels based on the information stored in the graph nodes and computes the similarity between pixels using Equation (12). This equation measures pixel similarity by calculating the color distance across RGB channels and the spatial distance in the $\left(x_{k},y_{k}\right)$ coordinates.

(12) $$\begin{array}{cc} C_{k} & ={\left[R_{k},G_{k},B_{k},x_{k},y_{k}\right]}^{T}\\ d_{\mathrm{RGB}} & =\sqrt{{\left(R_{k}-R_{i}\right)}^{2}+{\left(G_{k}-G_{i}\right)}^{2}+{\left(B_{k}-B_{i}\right)}^{2}}\\ d_{\mathrm{RGB}} & =\sqrt{{\left(x_{k}-x_{i}\right)}^{2}+{\left(y_{k}-y_{i}\right)}^{2}}\\ D_{s} & =d_{RGB}+\frac{m}{c}d_{xy} \end{array}$$

The parameters for the Felzenszwalb and DBSCAN algorithms were empirically determined through a grid search on a validation set to achieve the best balance between segmenting the gesture region completely and minimizing over-segmentation of noise. The final chosen parameters are listed in Table 1.

**Figure 6:**
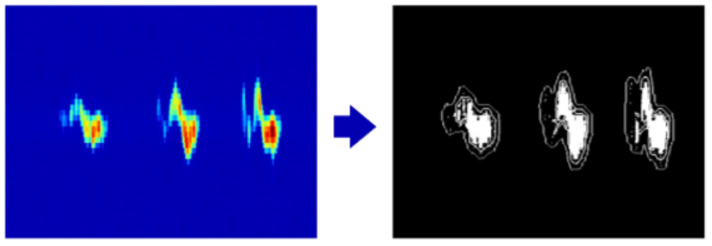
Segmentation of gesture feature regions using the Felzenszwalb algorithm.

This helps prevent the generation of excessively small regions, and the parameter is typically set to an appropriate value to avoid over-segmentation. The Felzenszwalb algorithm performs region merging in ascending order of dissimilarity based on pixel similarity. Two adjacent regions are merged only if their dissimilarity is below a predefined threshold. As illustrated in Figure 6, the Felzenszwalb algorithm effectively segments the gesture feature regions from the Doppler spectrogram.

**Figure 7:**
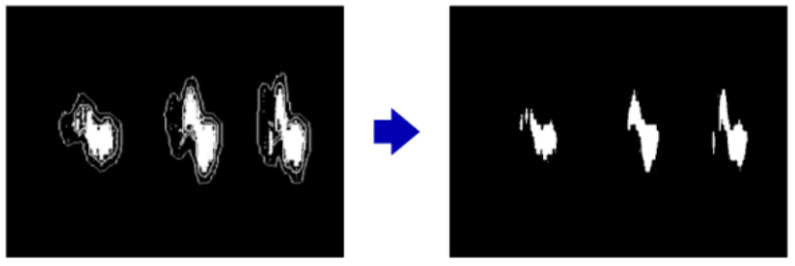
Noise reduction and segmentation refinement using the DBSCAN algorithm.

Due to the presence of fine-grained noise in Doppler images, the Felzenszwalb segmentation algorithm may incorrectly segment regions that do not belong to the actual gesture features. To mitigate errors caused by over-segmentation, this study incorporates the Density-Based Spatial Clustering of Applications with Noise (DBSCAN) algorithm [36] to refine and smooth the segmentation boundaries. DBSCAN clusters data points into dense regions while identifying outliers in sparse regions as noise. By defining a minimum neighborhood radius, data points are categorized as core points, border points, or noise points. Through the tuning of the radius parameter (Eps) and the minimum number of samples (MinSamples), the cluster with the lowest mean intensity is identified. The maximum value within this cluster is used as a dynamic threshold to eliminate fragmented segments. As shown in Figure 7, DBSCAN reduces invalid segmented areas and improves the overall segmentation quality.

### 3.3. Doppler Feature Extraction Algorithm

After segmenting the continuous gesture spectrogram, a segmentation mask is obtained. The segmentation mask is copied to form a three-channel mask, and the original image is multiplied by the matrix mask to obtain the gesture feature spectrogram. Using the segmentation mask, the Doppler extraction algorithm extracts the start time and end time of the gesture. To extract the time–frequency-domain features of the gesture, traverse the x-axis direction of the segmented grayscale image, mark the columns where there are segmentation pixels in the image, and save the x-values corresponding to these segmentation columns. Set a threshold (*T*) and traverse *T* frames forward; if there is no x value of the segmentation frame in these frames, it is used to distinguish the start time of different gestures, denoted as $t_{\mathrm{start}}$. Traverse backwards through *T* frames. If there is no x value for the segmentation frame in these frames, use it to distinguish the end times of different gestures, denoted as $t_{\mathrm{end}}$.

**Figure 8:**
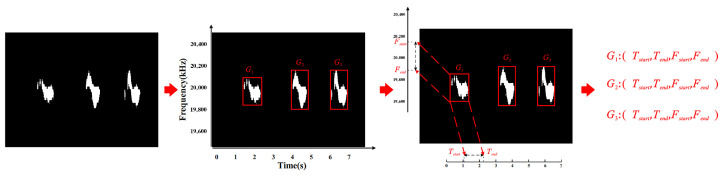
Time feature extraction.

Similarly, by traversing in the y-axis direction, we can obtain the maximum and minimum frequencies of the gestures. We sort the gestures according to the x-axis direction, calibrate the occurrence sequence of the gestures, and calibrate each gesture using two points (x,y), where *n* represents the number of gestures and the difference between *x* and *y* under the same *n* represents the time–frequency-domain range of the gesture, as shown in Figure 8.

**Figure 9:**
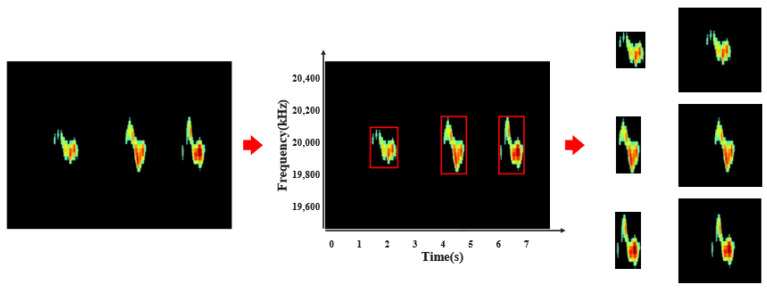
Gesture feature extraction.

Crop the anti-segmentation image based on the calibration points of each gesture and extract the gesture sequence number from the calibration points to obtain a dedicated anti-segmentation feature map for each gesture. By traversing the feature map, the frequency bandwidth and corresponding amplitude values in each sampling frame can be extracted; then, the image can be reshaped into a 224×224 input image. In this way, we decouple a single gesture feature map of multiple continuous gestures. This feature map significantly reduces the impact of environmental noise and preserves micro features such as user gesture initiation and release actions, enabling the classification model to better identify relevant features of different users and reduce the possibility of model overfitting, as shown in Figure 9.

### 3.4. Data Augmentation Based on User Gesture Information

In the field of ultrasonic gesture recognition, data augmentation technology is of great significance in improving the generalization ability and robustness of models. In the data augmentation section, SonarGS adopts a two-stage data augmentation scheme to expand the dataset. In the first stage, the feature information of user gestures is obtained through time–frequency-domain analysis in the Doppler feature extraction algorithm, and the time–frequency-domain mean of each gesture is calculated, thereby establishing gesture profiles for each gesture of different users. In order to further enrich the dataset, four stretching and translation transformation methods are adopted in the second stage of data augmentation. By performing x-axis stretching transformation on time–frequency-domain features, with stretching ratios ranging from 0.8 to 1.5 times, simulating gesture actions at different speeds, more diverse gesture data can be generated. By stretching the y-axis with stretching ratios ranging from 0.8 to 1.5 times, gestures with different action amplitudes can be simulated. By translating gestures in the x-axis direction, gestures with different start and end times are simulated, and by overlapping non gesture areas, multiple continuous gestures can be simulated with a single gesture. Through a two-stage data augmentation algorithm, more continuous multi-gesture samples with different speeds, times, amplitudes, and quantities can be generated, thereby enhancing the model’s ability to recognize various gesture changes and improving the overall performance of the system.

### 3.5. Identity Recognition Strategy

To ensure the accuracy and feasibility of recognition, SonarGS adopts a two-stage recognition strategy. In general, a classifier for the same person not only needs to provide gesture recognition results but also a confidence level higher than the set threshold. Differences in hand gestures and movements among individuals are related to their skeletal muscles and behavioral habits. For this purpose, we trained an independent model for each individual and transmitted the trained model weights to mobile devices through transfer learning. Due to the fact that single-gesture classification verification can be regarded as an independent prediction task, a confidence threshold is set to measure whether the device is unlocked. Only when the confidence level of the recognition result is higher than the set threshold will it be output as unlocked successfully.

Given that the model needs to run on a mobile device NPU and the classification model requires minimal memory and CPU resources, SonarGS adopts ShufflenetV2 as the classification network. Unlike traditional direct grouping convolution, ShufflenetV2 performs convolution grouping by randomly shuffling image channels, thereby improving the recognition rate of image classification. Therefore, our classifier is mainly based on the architecture design of ShufflenetV2. As a lightweight model, ShufflenetV2 cannot be measured solely by metrics such as floating-point operations (FLOPs) to evaluate its speed and accuracy, nor can it be evaluated solely by direct metrics such as accuracy. Experimental evidence shows that memory consumption time is also an important indicator affecting the deployment speed of the model in terms of inference time consumption. Therefore, based on the four guiding principles for efficient network design proposed in the original paper in which ShufflenetV2 was introduced [37], a gesture classification model was built to ensure that the model can achieve good classification performance while maintaining high efficiency.

(13) $$MAC=\left\{\begin{array}{c} hw\left(c_{1}+c_{2}\right)+c_{1}c_{2}\\ B=hwc_{1}c_{2} \end{array}\right.$$

where *B* Indicates the number of floating-point operations (FLOPs) required when using a 1 × 1 convolutional layer. Among them, *h* is the height of the input feature matrix, *w* is the width of the input feature matrix, $c_{1}$ is the number of channels in the input feature matrix, $c_{2}$ is the number of channels in the output feature matrix, and $c_{1}c_{2}$ represents the memory consumption of the convolution kernel. According to the arithmetic mean inequality (14),

(14) $$\frac{c_{1}+c_{2}}{2}\geq \sqrt{c_{1}c_{2}}.$$

By combining Equations (13) and (14), Equation (15) can be obtained as

(15) $$MAC\geq 2\sqrt{hwB}+\frac{B}{hw}.$$

Therefore, when the input feature matrix and output feature matrix of the convolutional layer are equal, the memory (multiply accumulate operation) reaches its minimum. Due to the equal numbers of input and output matrix channels in the network structure, the convolutional layer consumes the least amount of memory resources. As shown in Equations (16) and (17), assuming the number of floating-point operations (FLOPs) remains constant, as shown in Equation (18), as the number of integral groups in the test paper increases, the amount of memory multiplication and accumulation operations also increases. The more branch structures the model has, the longer the waiting time. In the model architecture, the input feature matrix is first divided into two branches, with no convolution operation performed on the shortcut branch and the same number of input and output channels on the other branch. Simultaneously. 1 × 1 convolution is used instead of group convolution to reduce memory consumption. Finally, when merging branches, concatenation is used instead of summation to ensure consistent input and output.

(16) $$MAC=hw\left(c_{1}+c_{2}\right)+\frac{c_{1}c_{2}}{g}$$

(17) $$B=\frac{hwc_{1}c_{2}}{g}$$

(18) $$MAC=hwc_{1}+\frac{Bg}{c_{1}}+\frac{B}{hw}$$

**Figure 10:**
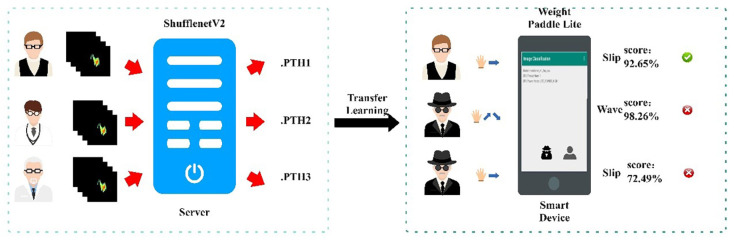
Identity recognition.

Given the complex and diverse micro features of each person’s gestures and the increasing number of gestures, the differences in gesture features between different individuals become more significant. Based on this principle, SonarGS extracts micro gesture features to classify gestures and achieve identity recognition. As shown in Figure 10, first, we trained a dedicated classification network for each person and input the gesture features extracted from the feature extraction module into the neural network for model training. When the accuracy of the validation set is high enough, model training is considered to be complete, and the model weight file (.pth) is sent to the mobile device. The inability of eavesdroppers to steal data based on a single weight file ensures the security of the information. We use the Paddle Lite lightweight framework to identify personnel identity on mobile devices through transfer learning. After data processing and feature extraction, the single sampling ultrasound signal is sent to the neural network on the mobile end to test the gesture feature area. Secondly, since the model is trained based on data from a single individual and their augmented data and for a single set of gestures, all gestures are correctly predicted as positive samples (Positive), while when any gesture is incorrectly predicted as negative, only the true-positive (TP) sample is correctly predicted as positive. Through this method, we can determine whether the intruder is a legitimate user and unlock the device after confirming its correctness.

## 4. Experiment and Analysis

### 4.1. Data Collection

This experiment collected data using five smartphones over a period of two months, including a Huawei P30 Pro (Huawei Technologies Co., Ltd., Shenzhen, China), Redmi K50 (Xiaomi Corporation (Redmi is its sub-brand), Beijing, China), iPhone 13 (Apple Inc., Cupertino, CA, USA), Redmi K60 (Xiaomi Corporation, Beijing, China), and OnePlus 9 Pro (OnePlus Technology Co., Ltd., Shenzhen, China). We invited 18 volunteers (9 males and 9 females) to participate in the experiment and asked them to develop an unlock password consisting of 0–9 gestures and five predetermined gestures. In addition, volunteers needed to complete a gesture password consisting of multiple single gestures within 8 s and record the start and end times of the gestures (with an error of 0.1 s). During the experiment, the user stood or sat still at a distance of 0.4 to 1 m from the device, keeping their torso relatively still, and moved their hands within a detection range of 0.8 m to perform gestures. At the same time, the observer annotated the start and end times of the landing hand gestures during the data collection process. In the model training dataset used in this study, gesture collectors complete gestures at three different speeds (fast, normal, and slow) and perform gesture actions at a position centered on the phone’s central axis and at an angle of $0^{\circ }$, with the gesture action no more than 0.4 m away from the phone. In the robustness experimental dataset used in this study, experimental datasets with different volumes, distances, and various environmental interferences were designed to verify the robustness of the system. We designed a non-standard operation robustness experimental dataset and a non-independent robustness experimental dataset for different devices and users, collecting a total of 5787 real gesture audio samples, which encompass a total of 16,609 gestures.

### 4.2. Deployment and Training Environment

The prototype of the SonarGS system consists of two main parts: one is an acoustic data acquisition module based on the WeChat mini program, and the other is a validation system deployed on the server side. As a data collection module, the WeChat mini program provides users with a convenient interface to collect and transmit acoustic signal data to the server in real time. The server-side verification system is responsible for processing, analyzing, and verifying the received data to complete core functions such as user identity authentication. In order to support efficient system operation and large-scale data processing, the server is equipped with a powerful hardware configuration, including one NVIDIA GeForce GTX 1080Ti GPU, 32 GB of memory, and an Intel Core i9-7900X 3.3 GHz CPU. This configuration not only meets the training requirements of complex deep learning models but also quickly responds to validation requests from front-end devices, ensuring the real-time performance and accuracy of the system. During the testing phase, we deployed the server-side trained model (stored in .psh file format) on a smartphone using the PaddleLite framework. PaddleLite, as a lightweight deep learning inference framework, can efficiently run models while minimizing the use of device hardware resources. The lock screen of smart devices is simulated using a mobile program developed with Android Studio to achieve edge deployment of the model.

### 4.3. Experimental Evaluation

#### 4.3.1. Single-User, Single-Gesture Classification Evaluation

**Figure 11:**
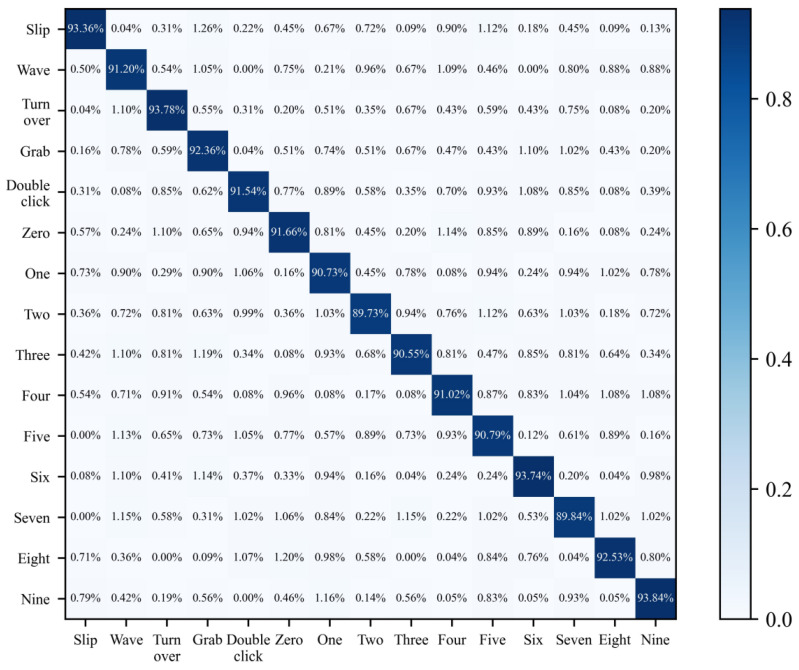
Confusion matrix for single-gesture classification.

In order to achieve accurate classification of gestures, it was necessary to verify the accuracy of the model in classifying fifteen gesture actions. This experiment evaluated the model using an independent test dataset. In the experimental design, based on the data collected from 18 volunteers (including 9 males and 9 females), the dataset was divided into a training set and a testing set, with a ratio of 80% to 20% respectively, to evaluate the model’s ability to classify a single user and gesture. As shown in Figure 11, a confusion matrix containing fifteen gestures was obtained, which detailed the classification results of the model on the test set. The experimental results showed that the average recognition accuracy of the model for gestures was about 91.58%. This result indicates that the ShufflenetV2 model performs well in single-gesture category recognition tasks and can effectively recognize target gestures from the background.

#### 4.3.2. Evaluation of Single-User, Multi-Gesture Segmentation and Recognition Ability

**Figure 12:**
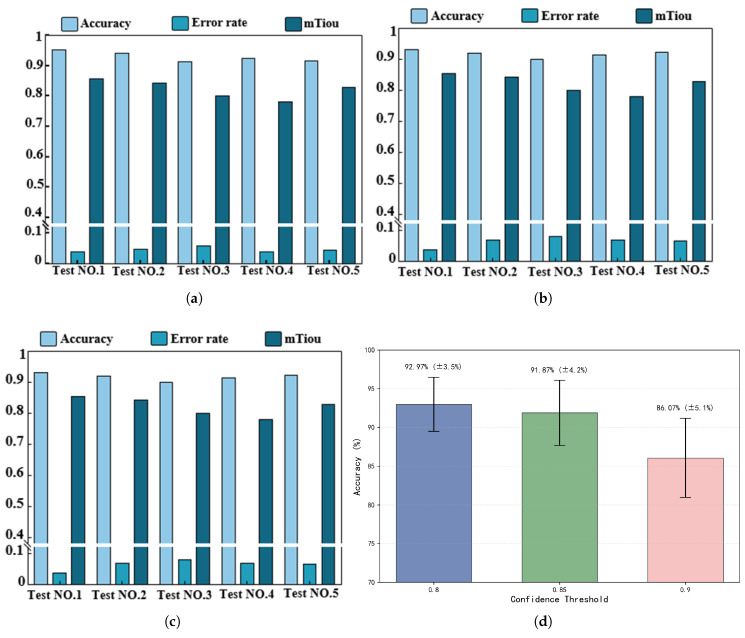
Bar chart of single-user, multi-gesture evaluation. (**a**) Low threshold (0.8); (**b**) medium threshold (0.85); (**c**) high threshold (0.9); (**d**) recognition accuracy at different thresholds (The error bar indicates +1 standard deviation, m=18).

In order to systematically evaluate the segmentation and recognition ability of the model for multiple continuous gestures, *m* independent models were trained to ensure that the models can adapt to the gesture features of different users. This experiment set three thresholds (0.8, 0.85, and 0.9), and the system recognized each individual gesture in sequence and judged recognition success when the predicted confidence was greater than the threshold. This experiment selected five participants (three males and two females) and tested the model using their corresponding weight files. These weight files are trained based on the user’s own data and can reflect the user’s unique behavioral characteristics and gesture patterns. The accuracy rate is calculated by dividing the number of experiments where each gesture recognition is correct and the confidence level is above the threshold by the total number of experiments. The error rate is calculated by dividing all experiments that were not successfully judged by the total number of experiments. Due to the inability to directly measure precise frequency-domain values, in this experiment, only the segmentation effect of the temporal attributes of gestures was evaluated. Time-domain intersection and comparison were used to evaluate the model’s ability to segment gestures. As shown in Figure 12, the experimental results demonstrate the performance differences of the model under different confidence thresholds. When the confidence threshold is 0.85, the average accuracy of the model is about 91.87%, the error rate is about 8.13%, and the time-domain intersection ratio is about 0.82%. This indicates that at a moderate confidence threshold, the model can balance accuracy and error rates well while maintaining high temporal consistency. At a confidence threshold of 0.90, the accuracy is approximately 86.07%, the error rate is approximately 13.92%, and the time-domain intersection-to-union ratio is approximately 0.82. When the confidence threshold is 0.80, the accuracy is the highest (about 92.97%), the error rate is below 7.03%, and the time-domain intersection-to-union ratio is about 0.82. Experimental results have shown that the system can achieve high accuracy and a low segmentation error rate for continuous multi-handed password recognition and has excellent time-domain segmentation properties.

#### 4.3.3. Data Enhancement Evaluation

**Figure 13:**
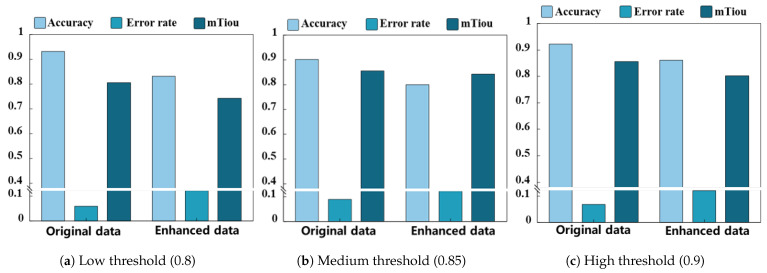
Bar chart assessing data enhancement capability.

To validate the effectiveness of data augmentation, this experiment randomly selected 100 data points from the original dataset and matched them with 100 corresponding data points from the augmented dataset. The same model weights were used to calculate the average accuracy rate, error rate, and time intersection ratio (Tiou) for both datasets. The experimental results are shown in Figure 13. At a confidence threshold of 0.80, the accuracy rate of the original dataset was 95.23%, the error rate was 3.90%, and the Tiou was 0.85, while the accuracy rate of the data-enhanced dataset was 93.12%, the error rate was 6.87%, and the Tiou was 0.84. At a confidence threshold of 0.85, the accuracy rate of the original dataset was 97.22%, the error rate was 1.94%, and the Tiou was 0.85; the accuracy rate of the data augmentation group was 92.12%, with the error rate not explicitly provided, and the Tiou was 0.84. At a confidence threshold of 0.90, the accuracy rate of the original dataset was 90.32%, the error rate was 3.07%, and the Tiou was 0.85; the accuracy rate of the data augmentation group was 88.21%, the error rate was 6.87%, and the Tiou was 0.84. The experimental results indicate that the two-stage data augmentation method proposed in this paper can enrich the dataset without altering the microscopic features of the gestures and can be effectively applied to data augmentation tasks.

#### 4.3.4. Robustness Testing

In practical applications, different distances between smart devices and users can have varying degrees of impact on Doppler spectrum diagrams, thereby affecting the accuracy of verification. Therefore, in this experiment, samples collected at distances of 60 cm and 80 cm were tested using a single basic model.

**Figure 14:**
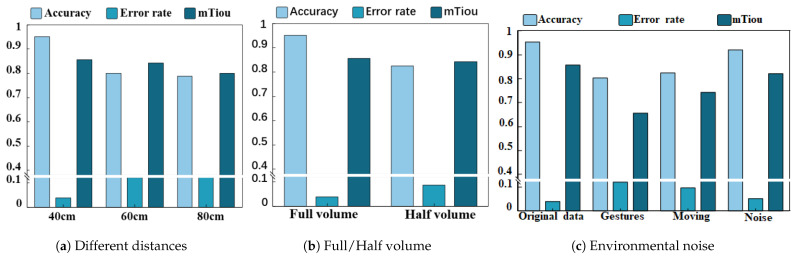
Bar chart assessing system robustness.

As shown in Figure 14a, the results indicate that SonarGS achieves an accuracy rate of 85.23% at 60 cm, an error rate of 11.95%, and a time-domain intersection ratio (Tiou) of 0.85; at 80 cm, the accuracy rate is 78.98%, the error rate is 19.07%, and the Tiou is 0.84. The experimental results demonstrate that SonarGS can still correctly recognize most gestures and accurately distinguish between different users at different operating distances, with no significant reduction in detection accuracy. Similarly, playing ultrasonic carriers at different volumes also affects SonarGS. Therefore, models were trained using data collected at full volume and half volume. As shown in Figure 14b, when the volume is incomplete, the accuracy rate is 82.56%, the error rate is 8.79%, and the Tiou value is 0.84. The experimental results indicate that incomplete volume does have a certain impact on SonarGS, but this impact is within an acceptable range. Additionally, we tested the effects of personnel movement, gesture interference, and noisy environments on SonarGS, as shown in Figure 14c. In an environment with personnel movement, the accuracy rate was 81.69%, the error rate was 13.97%, and the Tiou value was 0.74; in environments with gesture interference, the accuracy rate was 80.22%, the error rate was 13.90%, and the Tiou value was 0.65; and in noisy environments, the accuracy rate was 92.45%, the error rate was 5.03%, and the Tiou value was 0.82. The experimental results indicate that SonarGS has strong resistance to noise and can still achieve good recognition results, even in environments with gesture interference and human interference. SonarGS demonstrates excellent robustness under various distances, volumes, and complex environmental conditions, effectively addressing multiple interference factors in real-world applications and providing users with stable and reliable gesture recognition and identity verification functions.

Furthermore, we preliminarily tested the system in an outdoor environment with sunlight interference and in the presence of a strong ultrasonic source (a 20 kHz tone played from another phone). The accuracy dropped by 10–15% in these extreme conditions, primarily due to an increased noise floor and interference. Future work will focus on enhancing the algorithm’s robustness in such challenging scenarios.

#### 4.3.5. Five-Fold Cross-Over Experiment Identity Verification

To comprehensively evaluate the performance of the SonarGS system, this experiment employed a five-fold cross-validation method to verify the security of the SonarGS device-unlocking process. To maximize the validation of model performance, this experiment used the same set of gesture passwords. Five volunteers (three males and two females) were invited, with each volunteer using the same device to perform the same set of continuous gesture actions, thereby generating independent datasets. Based on these data, five classification models were trained, with each model corresponding to a volunteer’s dataset, resulting in five datasets and five sets of model weights. This experiment combined the five models with the five datasets using a Cartesian product, generating 25 different model–dataset combinations. During the testing phase, each weight model was used to predict the continuous multi-gesture passwords issued by different users. Authentication was deemed successful when each single gesture category was correctly predicted and the prediction confidence exceeded the threshold. The number of successful authentications divided by the total number of attempts represents the probability of the current user successfully unlocking the smart device under the current model.

**Figure 15:**
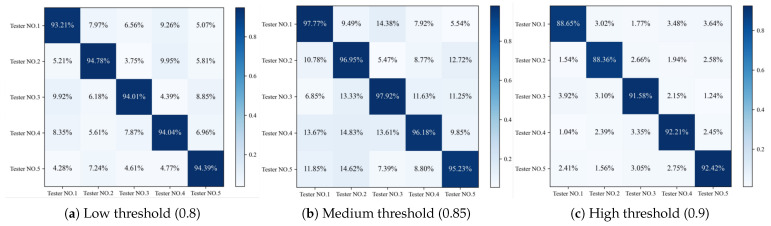
Five-fold cross-validation heat maps.

Figure 15 displays the heat maps of five-fold cross-validation at different confidence thresholds (0.8, 0.85, 0.9). The diagonal lines in the heat maps represent the probability of successful identification of experimental subjects by the classification models of the five experimenters. The horizontal axes of the heat maps indicate the unlocking probability of the same experimenter under different models, while the vertical axes indicate the unlocking probability of the same classification model across different user datasets. The experimental results show that when the threshold is 0.8, the minimum accuracy of correct recognition is 95.23%, but the maximum false recognition rate is 14.62%. When the threshold is 0.85, the minimum accuracy of correct recognition is 93.23%, and the maximum false recognition rate is 9.95%. When the confidence threshold is 0.9, the lowest accuracy rate for correct recognition is 88.36%, while the false recognition rate is extremely low, with a maximum of 3.92%. This indicates that at higher confidence thresholds, the model can recognize gestures with extremely high precision while almost completely avoiding false recognition. This result indicates that while lower thresholds can improve the model’s recognition rate, they may also lead to more false positives. Experimental results show that as the confidence threshold increases, the probability of correct recognition increases, while the probability of false recognition decreases. Additionally, performance varies across different datasets at different thresholds, but all threshold settings can accurately distinguish users through gestures. This finding indicates that by reasonably setting the confidence threshold, the SonarGS system can achieve high-precision gesture recognition across different datasets and user conditions, thereby providing reliable technical support for user authentication in practical applications.

#### 4.3.6. Comparison of the Performance of Statistical Tests with That of Other Methods

We conducted several statistical tests for the experiments of interest using the SciPy and statsmodels packages in Python 3.9. The Shapiro–Wilk test was employed to assess normality, while Levene’s test was used to test the equality of variances. For data that did not meet the assumptions required for parametric tests, we utilized the appropriate non-parametric tests. The significance level was set at α=0.05, and we applied Bonferroni correction for multiple comparisons. The results are presented in Table 2.

As can be seen, a repeated-measures ANOVA revealed a significant main effect of the confidence threshold on accuracy (F(2,8) = 15.37, *p* = 0.002, $\eta^{2}=0.79$). Post hoc paired *t*-tests indicated that accuracy at a threshold of 0.9 was significantly lower than at thresholds of 0.8 (t(4) = 5.23, *p* = 0.006) and 0.85 (t(4) = 4.17, *p* = 0.014). However, the difference between thresholds of 0.8 and 0.85 was not significant (t(4) = 1.42, *p* = 0.23). Additionally, a paired *t*-test showed a significant difference in accuracy at distances of 60 cm compared to 80 cm (t(17) = 3.89, *p* = 0.001, Cohen’s d = 0.92), suggesting that the operating distance significantly impacted system performance. Overall, the statistical analyses demonstrated that the choice of confidence thresholds significantly affected system performance. While the lower threshold of 0.8 yielded the highest accuracy, the difference with the medium threshold of 0.85 was not significant. However, when the threshold was raised to 0.9, accuracy decreased significantly. This decrease may be attributed to the overly strict threshold filtering out some correct recognition results. These findings provide empirical evidence for the selection of appropriate thresholds in practical applications.

To evaluate the overall performance of the method presented in this paper, we compared it with existing gesture authentication systems, as illustrated in Table 3. The results indicate that the proposed method demonstrates high feasibility and usability.

Furthermore, by analyzing the computational efficiency, the entire authentication pipeline, including signal processing, segmentation, feature extraction, and model inference, was deployed on a Redmi K50 smartphone for performance evaluation. The average processing time for an 8-second audio sample is approximately 1.2 s, which is significantly faster than the gesture duration and demonstrates the feasibility of real-time authentication. The majority of the time is consumed by the STFT and segmentation steps, while the ShuffleNetV2 inference on the NPU takes less than 50 ms.

## 5. Conclusions and Discussion

This study introduces SonarGS, an intelligent device gesture unlocking system based on ultrasonic gesture recognition and unsupervised segmentation. User identity is determined and the device unlocked by analyzing and extracting the time–frequency-domain range of the user’s continuous multi-gestures and the combination of gesture categories. To this end, this study proposes an unsupervised segmentation algorithm that precisely segments the pixel-level boundaries of gesture feature regions and extracts time–frequency-domain information and gesture feature regions using a Doppler feature extraction algorithm. This paper introduces a two-stage data augmentation method to generate a dataset matching the user’s gesture habits based on a small number of samples. By training a gesture classification model for each user on the server side and implementing edge deployment via PaddleLite, the system uses transfer learning to recognize gesture passwords. SonarGS not only enhances user experience but also ensures data security. Experimental results show that SonarGS demonstrates excellent recognition capabilities in five-fold cross-validation experiments, with an average accuracy rate of up to 93.56% across three different confidence thresholds. Robustness experiments and replay experiments confirm that SonarGS possesses a high degree of adaptability to complex environments and resistance to replay attacks. Experiments indicate that SonarGS can provide users with a secure, efficient, and reliable device-unlocking solution in practical applications.

This paper has conducted relevant research on the recognition of continuous multi-gestures based on acoustic signals, but there are still some issues that require further research and discussion in the future:

1. Application of large language models and multi-agent systems in gesture recognition: The use of large language models in the field of wireless sensing is a new research direction. Through the application of thought chains, fine-grained segmentation and recognition of gestures have become a new research direction. Therefore, future research will explore how to use fine-tuned large language models to reduce noise, segment and recognize multiple continuous gestures, and seek the best research solutions. We envision using a fine-tuned LLM not for direct gesture recognition but as a powerful semantic engine to guide the segmentation and feature extraction process. For example, the LLM could be prompted to generate a “chain of thought” that outlines the optimal steps for analyzing a complex gesture sequence, potentially improving the segmentation algorithm’s adaptability to new, unseen gesture patterns.
2. Development of an embedded edge computing acoustic development board: The development of an embedded edge computing acoustic development board is a critical step in achieving continuous multi-gesture recognition. While progress has been made in acoustic signal processing and gesture recognition algorithms, challenges remain in hardware development. Future research will focus on developing low-power, high-performance embedded development boards to meet the requirements of real-time gesture recognition. Additionally, the development board must possess robust computational capabilities to support complex signal processing and machine learning algorithms, thereby providing strong support for the widespread application of continuous multi-gesture recognition technology. To overcome the heterogeneity of commercial smartphones, we propose the development a dedicated low-power, high-performance embedded acoustic sensing board. This board would standardize the ultrasonic transceiver hardware, ensuring consistent signal quality. Our proposed SonarGS algorithm would be deployed directly on this board’s processor, creating a turnkey solution for secure gesture authentication that can be integrated into various IoT devices beyond smartphones.
3. A potential limitation of this study is the use of smartphones from a limited number of manufacturers. Although our system showed robustness across the five tested models, generalizability to all smartphone models cannot be guaranteed due to possible variations in speaker and microphone frequency response characteristics. This is an important aspect for future large-scale testing.

## Figures and Tables

**Table 1:** Parameter selection for algorithms.

Algorithm	Parameter	Value	Reasons for Selection
Felzenszwalb	Scale	500	Control of the superpixel size; the larger the value, the larger the area. This value can effectively merge small noise areas.
	Sigma	0.8	Color similarity standard deviation, used for Gaussian smoothing. This value can moderately smooth out small noise points in the spectrogram.
	Minsize	100	Minimum-area pixel count to prevent the generation of meaningless fragments that are too small.
DBSCAN	Eps	5	Neighborhood radius, used to determine the core point, based on the average distance setting of the segmented region pixels.
	MinSamples	15	The minimum number of samples required to form the core point. This value can effectively filter out small noise clusters.

**Table 2:** Statistical comparison results under different conditions. The number of asterisks represents the level of significance; the more asterisks, the higher the level of significance and the more reliable the results. * *p* < 0.05, ** *p* < 0.01, *** *p* < 0.001, n.s. not significant.

Comparison Group	Testing Method	Statistical Measure	*p*-Value	Effect Size	Significance
Threshold 0.8 vs. 0.85	paired *t* test	t(4) = 1.42	0.23	Cohen’s d = 0.63	n.s.
Threshold 0.8 vs. 0.9	paired *t* test	t(4) = 5.23	0.006 **	Cohen’s d = 2.34	**
Threshold 0.85 vs. 0.9	paired *t* test	t(4) = 4.17	0.014 *	Cohen’s d = 1.86	*
60 cm vs. 80 cm	paired *t* test	t(17) = 3.89	0.001 **	Cohen’s d = 0.92	**
Full volume vs. Half volume	paired *t* test	t(17) = 4.52	<0.001 ***	Cohen’s d = 1.07	***

**Table 3:** Performance comparison with existing gesture authentication systems.

System Name	Sensing Mode	Core Method	Authentication Method	Accuracy	Resistance to Replay Attacks
SonarGS (Ours)	Ultrasound	Unsupervised segmentation + ShuffleNetV2	Continuous Gesture	94.56%	Yes
Reference21	Ultrasound	Spectral Feature Analysis	Lip Language	89%	Unassessed
Reference24	WiFi CSI	Deep Learning	Continuous Gestures	94%	Unassessed

## Data Availability

Data analyzed during this study are included in this article.
